# A new leafmining pest of guava: *Hesperolyra guajavifoliae* sp. nov., with comments on the diagnostics of the endemic Neotropical genus *Hesperolyra* van Nieukerken (Lepidoptera, Nepticulidae)

**DOI:** 10.3897/zookeys.900.46332

**Published:** 2019-12-31

**Authors:** Jonas R. Stonis, Andrius Remeikis, Arūnas Diškus, Svetlana Orlovskytė, Sergio A. Vargas, Maria Alma Solis

**Affiliations:** 1 Institute of Ecology, Nature Research Centre, Akademijos St. 2, Vilnius LT-08412, Lithuania Nature Research Centre Vilnius Lithuania; 2 Jardín Botánico de Bogotá José Celestino Mutis, Av. Calle 63 No. 68-95, Bogotá, Colombia Jardín Botánico de Bogotá José Celestino Mutis Bogota Colombia; 3 Laboratorio de Entomología, UNESIS, Departamento de Biología, Pontificia Universidad Javeriana, Carrera 7, No. 43-82, Bogotá, Colombia Pontificia Universidad Javeriana Bogota Colombia; 4 Systematic Entomology Laboratory, ARS, USDA, National Museum of Natural History, Smithsonian Institution, Washington, D.C., 20013-7012, USA National Museum of Natural History, Smithsonian Institution Washington United States of America

**Keywords:** New species, pygmy moths, guayaba, *Psidium guajava*, leaf mines, taxonomy, Colombia

## Abstract

We describe a new pest of guava (*Psidium guajava* L.), *Hesperolyra guajavifoliae* Stonis & Vargas, **sp. nov.**, that was recently discovered in western Colombia. *Hesperolyra* van Nieukerken is a small, Neotropical genus of pygmy moths (Nepticulidae). We re-examine and document the complex morphology of the male genitalia of the generic type species, *H. diskusi* (Puplesis & Robinson). We discuss the diagnostics and composition of the genus and provide a simple pictorial differentiation scheme for all currently known representatives of the genus. The new species is illustrated with photographs of the adults, some of the immature stages, male and female genitalia, and leaf mines. A link to the COI barcodes of *H. guajavifoliae***sp. nov.** is provided and the relationship of *Hesperolyra* to other genera is discussed.

## Introduction

Guava or guayaba (*Psidium guajava* L.) is an important shrub or small tree cultivated for its fruit in many tropical countries in Asia, Africa, South America and the Caribbean. The fruit can be eaten raw or cooked, but, it is mostly known for its processed fruit products, and can be an integral part of local and international cuisine. The leaves and fruits are also fed to livestock (Heuzé et al. 2017). In some countries, this plant is also used in local traditional medicine (ethnopharmacology) to deal with numerous medical issues such as inflammation, diabetes, hypertension, tooth decay, wounds, ulcers, fever, diarrhea, lung ailments, rheumatism, and as a pain-relief remedy (Gutiérrez et al. 2008). Guava usually grows in areas below 1000 m, annual mean temperatures ranging from 23 to 28 °C, and 1500–2000 mm rainfall (Heuzé et al. 2017). In some tropical areas the plant can occur at altitudes up to 2000 m, in temperatures of 15–45 °C, and quiescent trees can even survive light frosts (Heuzé et al. 2017). Although the origin of this widespread species is not clear, it is believed to be native to countries in tropical America (Germplasm Resources Information Network 2019).

**Figures 1–6.**
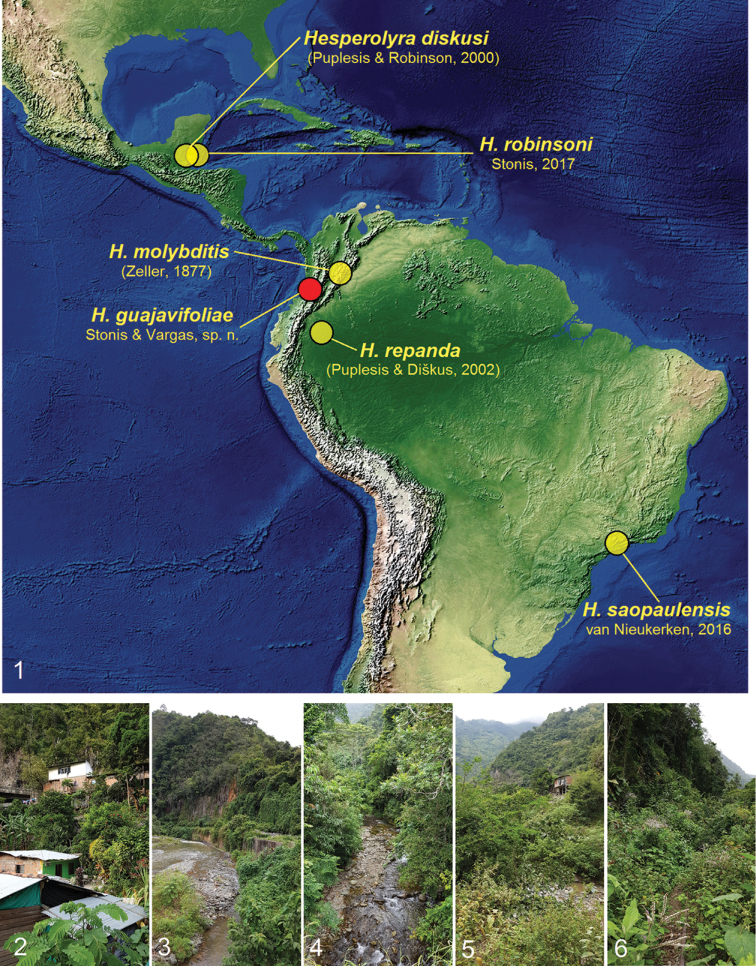
Distribution map of currently known *Hesperolyra* species and habitat of *H. guajavifoliae* Stonis & Vargas sp. nov. **1** Distribution map (the map base, courtesy of Tom Patterson, USA) **2, 5, 6** El Naranjo, 3°46'46"N, 76°43'63"W, 550 m **3, 4** Cisneros, 3°46'27"N, 76°44'40"W, 450 m.

In late January to early March 2019, we conducted fieldwork in the Departamento de Valle del Cauca, northwest of Dagua in southwestern Colombia (Figs 2–6), where *Psidium guajava* is a common plant cultivated in orchards and widespread in the wild in various anthropogenic or natural habitats. We expected to find *Ozadelpha guajavae* (Puplesis & Diškus) (Lepidoptera, Nepticulidae), a guava-feeding nepticulid species described a few degrees south from the same western tropical slopes of the Andes in equatorial Ecuador (Puplesis et al. 2002a). It was later recorded in large numbers in the Andes of southern Ecuador near the Peruvian border (Remeikis et al. 2014). However, during our fieldwork in western Colombia, we discovered another species producing leaf mines in mass quantities on *P. guajava*. It appeared to be a new and distinctive species belonging to the recently erected, Neotropical genus *Hesperolyra* van Nieukerken. Including the newly discovered species described below, *Hesperolyra* now comprises six species that occur from Central America to the Atlantic coast of Brazil. Prior to our study, *H. molybditis* (Zeller, 1877), of which the host plant is unknown, was known to occur in central Colombia (Fig. 1). Previously, only one species of *Hesperolyra* had host-plant family data; *H. saopaulensis* van Nieukerken 2016 was reared from an unidentified Myrtaceae plant (van Nieukerken et al. 2016b).

Nepticulidae , or pygmy moths, are miners (occasionally gall inducers, e.g., van Nieukerken et al. 2016b) of assimilative tissues of plants. Some species have been included on lists of cultivated plant pests (e.g., Kuznetzov and Puplesis 1994). A general characterization of this family was provided by several authors, notably Scoble (1983), van Nieukerken (1986), Johansson et al. (1990), Puplesis (1994), Puplesis and Robinson (2000), Puplesis and Diškus (2003), Diškus and Stonis (2012), and recently van Nieukerken et al. (2016b). Nepticulidae are distributed worldwide and occur in almost all terrestrial habitats. Because of species endemism and a high degree of stenophagy, these tiny lepidopterans may serve as tools for monitoring the biodiversity richness of habitats and provide data on the evolutionary processes (Diškus and Stonis 2012; Remeikis 2017). Being some of the smallest moths, adults remain rare in many scientific collections, especially in the tropical countries of Latin America. However, in the field, leaf mines produced by nepticulid larvae are usually distinctive and easy to spot. Sometimes these leaf mines may appear in very large numbers, dramatically reducing the assimilative tissue of mined host plants. Sometimes hundreds or thousands of larvae may feed upon a single tree at the same time, as in the case of the Holarctic *Ectoedemia occultella* (Linnaeus) and East-Asian *E. picturata* Puplesis (Puplesis 1994).

Below, we provide a description of the new species, documentation of leaf mines, adults, and their male and female genitalia. We also provide comments on the diagnostics of *Hesperolyra*, with a simple pictorial tool for identification of the currently known *Hesperolyra* species.

## Material and methods

### Material

The material was collected in 2019 in the Valle del Cauca in Colombia by Jonas R. Stonis and Sergio A. Vargas. We were assisted by Franklin J. Galindo (Collecting Permit No. 2019007511-1-000 by *Autoridad Nacional de Licencias Ambientales*, Bogotá, Colombia). The material is deposited at the collection of the Laboratorio de Entomología, UNESIS, Departamento de Biología, Pontificia Universidad Javeriana, Bogotá, Colombia (**MPUJ**). Additional material of the type species *Hesperolyra diskusi* (Puplesis & Robinson), used for comparison and re-study of the complicated morphology of the male genitalia, is currently at the Lithuanian University of Educational Sciences, Vilnius, Lithuania (**LEU**) and will be transferred for permanent deposition to the collection of the Zoological Museum, Natural History Museum of Denmark, Copenhagen (**ZMUC**).

### Methods

We followed collecting methods and protocols for species description outlined in Johansson et al. (1990), Puplesis and Diškus (2003), and Stonis et al. (2016). After maceration of the abdomen in 10% KOH and subsequent cleaning, male genital capsules were removed from the abdomen and mounted ventral side up. Both male and female genitalia were mounted in Euparal. In most cases the phallus was severed from the genital capsule. Abdominal pelts and female genitalia were stained with Chlorazol Black (*Direct Black 38/Azo Black*), male genitalia were left unstained (for a detailed description of the used method see Stonis et al. 2014).

Permanent preparations on microscope slides were photographed and studied using a Leica DM2500 microscope and a Leica DFC420 digital camera. Adults were photographed using a Leica S6D stereoscopic microscope with attached Leica DFC290 digital camera, except for Figs 13–20, 23–27, which were photographed using a Lomo MBS10 stereoscopic microscope and temporary attached cellular telephone Samsung Galaxy S7 with a camera. The specimens were subjected to high intensity, daylight illumination and rotated to ascertain ground colour and reflection of the adult scaling.

The descriptive terminology of morphological structures follows Puplesis and Robinson (2000), except for the term “aedeagus”, which is here referred to as “phallus” and the term “cilia”, which is here referred to as “fringe”.

Molecular analysis. The fragment of the mitochondrial COI gene that includes the standard barcode region for the animal kingdom (Hebert et al. 2003) was analysed to evaluate the molecular distinctness of the newly described *Hesperolyra guajavifoliae* sp. nov. from closely related Nepticulidae species. To achieve this goal, eight specimens of *H. guajavifoliae* sp. nov., two specimens of *Acalyptris* Meyrick (Lepidoptera, Nepticulidae), and one specimen of *Pseudopostega* Kozlov (Lepidoptera, Opostegidae) were barcoded (Table 1). The total genomic DNA was extracted from legs or the whole specimens stored in 96% ethanol, using the GeneJet Genomic DNA Purification kit (Thermo Fisher Scientific Baltics) according to the manufacturer’s specifications. A 674 bp fragment of the mitochondrial COI gene was amplified with the primers T3Lep-f (5’-ATTAACCCTCACTAAAGTCWACHAATCATAAARATATTGG-3’; modified Lep-f1 (Hebert et al. 2004)) and T7Nancy-r (5’-AATACGACTCACTATAGGDARAATTARAATRTAAACYTCWG-3’; modified Nancy (Simon et al. 1994)). All PCR reactions were carried out in a volume of 25 µL containing 12.5 µL of 2× PCR buffer (Thermo Fisher Scientific Baltics), 2.5 µL of 10 pmol of each primer (Macrobio), 6.5 µL of deionised water, and 1 µL of genomic DNA. All amplification reactions were performed with the MasterCycler personal thermocycler (Eppendorf) with the following conditions: initial denaturation at 95 °C for 1 min; 45 cycles of denaturation at 94 °C for 40 s, annealing at 45 °C for 40 s, extention at 72 °C for 1 min; with a final extension at 72 °C for 5 min. PCR product quality was checked by electrophoresis on 1.5 % agarose gel (Thermo Fisher Scientific Baltics) stained with 10 000× GelRed (Biotium) and visualized under 305 nm UV light. The excess of primers and dNTPs was removed with exonuclease I and alkaline phosphatase (Thermo Fisher Scientific Baltics) prior to automatic sequencing by the BigDye Terminator v3.1 Cycle Sequencing Kit (Applied Biosystems) in Macrogen Inc. (Seoul, South Korea). The sequences were manually aligned using BioEdit 7.2.5 (Hall 1999). The final aligned length of the dataset was 657 bp. The shorter than expected (609 bp-long) sequence of *Acalyptris* sp. involved in the analysis was due to unsuccessful sequencing. All sequences obtained in this study have been deposited in the GenBank database (www.ncbi.nlm.nih.gov/Genbank) under the accession numbers provided in Table 1. In addition, previously published Nepticulidae sequences downloaded from the BOLD platform (Ratnasingham and Hebert 2007) (https://www.boldsystems.org) were involved in further analysis. The nucleotide-sequence divergence was calculated using the Kimura 2-parameter distance (Kimura 1980) model and graphically displayed in the Neighbour-Joining (NJ) tree by the MEGA 6 software (Tamura et al. 2013). Robustness of the inferred tree was evaluated by bootstrapping with 10,000 replicates; the distantly related *Pseudopostega* sp. was used as an outgroup. MEGA 6 was also used for the calculation of pairwise distances, the mean distances within and between species.

**Table 1.** Data of studied Lepidoptera specimens and their DNA barcodes.

Species	Sex	Locality	Coordinates	Date	Collector	Sample ID	Genbank accession
**Nepticulidae**:							
*Acalyptris bifidus* Puplesis & Robinson	♂	COLOMBIA, Valle del Cauca, El Naranjo	3°47’2”N, 76°43’14”W	21–23.ii.2019	J. R. Stonis & S. Vargas	AB2517	MN732881
*Acalyptris* Meyrick sp.	♀	COLOMBIA, Valle del Cauca, Lobo Guerrero	3°45’42’’N, 76°39’46’’W	8.ii–3.iii.2019	J. R. Stonis & S. Vargas	AC2521	MN732881
*Hesperolyra guajavifoliae* Stonis & Vargas, sp. nov.	♂	COLOMBIA, Valle del Cauca, Cisneros	3°46’27”N, 76°44’40”W	11.ii–3.iii.2019	J. R. Stonis & S. Vargas	HG2527	MN732873
	♀	COLOMBIA, Valle del Cauca, Cisneros	3°46’27”N, 76°44’40”W	11.ii–3.iii.2019	J. R. Stonis & S. Vargas	HG2528	MN732874
	♂	COLOMBIA, Valle del Cauca, Cisneros	3°46’27”N, 76°44’40”W	11.ii–3.iii.2019	J. R. Stonis & S. Vargas	HG2529	MN732875
	♂	COLOMBIA, Valle del Cauca, Cisneros	3°46’27”N, 76°44’40”W	11.ii–3.iii.2019	J. R. Stonis & S. Vargas	HG2530	MN732876
	♀	COLOMBIA, Valle del Cauca, Cisneros	3°46’27”N, 76°44’40”W	11.ii–3.iii.2019	J. R. Stonis & S. Vargas	HG2532	MN732877
	♀	COLOMBIA, Valle del Cauca, Cisneros	3°46’27”N, 76°44’40”W	11.ii–3.iii.2019	J. R. Stonis & S. Vargas	HG2534	MN732878
	♂	COLOMBIA, Valle del Cauca, Cisneros	3°46’27”N, 76°44’40”W	11.ii–3.iii.2019	J. R. Stonis & S. Vargas	HG2535	MN732879
	♀	COLOMBIA, Valle del Cauca, Cisneros	3°46’27”N, 76°44’40”W	11.ii–3.iii.2019	J. R. Stonis & S. Vargas	HG2536	MN732872
**Opostegidae**:							
*Pseudopostega* Kozlov sp.	♂	COLOMBIA, Valle del Cauca, SW of Cali, Vía Villa Carmelo, Desarrollo Biodiverso	none	29–30.i.2019	J. R. Stonis & S. Hill	PC2516	MN732882

## New species description

### Hesperolyra guajavifoliae

Taxon classificationAnimaliaLepidopteraNepticulidae

Stonis & Vargas sp. nov.

24CCB9EE-AC48-5C3C-8F9D-08B7F7090970

http://zoobank.org/C4224ABF-1778-4651-BC9F-E5E3A48A100D

#### Type-specimen.

***Holotype***: male, pinned, with genitalia slide no. RA1033. Original label: Colombia, Departamento de Valle del Cauca, Municipio de Dagua, Cisneros, 3°46'27"N, 76°44'40"W, 450 m, larva on *Psidium guajava*, fieldcard no. SV003, 11 Feb – 3 Mar 2019, J. R. Stonis and S. A. Vargas. (MPUJ).

#### Diagnosis.

Externally, adults of the new species are distinguishable from all other Neotropical Nepticulidae, including congeneric *Hesperolyra*, by a dark, oblique fascia and two small, dark, basal and apical spots on the forewing. However, in some specimens, including worn ones, the spots may be inconspicuous or absent. In the male genitalia, a large apical process of the valva, two large, horn-like processes fused with the transtilla and weakly developed cornuti in the phallus distinguish *H. guajavifoliae* sp. nov. from all other *Hesperolyra* species. In the female genitalia, the unique, large vaginal sclerite and distally wide vesicles of ductus spermathecae are hypothesized to be unique to this species, but this character may not remain valid for species differentiation because females of many nepticulid species are unknown and remain to be discovered. *Hesperolyra guajavifoliae* sp. nov. is distinguishable from another guava feeder, *Ozadelpha guajavae* Puplesis & Robinson, by a dark, oblique fascia and two small spots on the forewing of the adults, and by blotch-like leaf mines (leaf mines of *O. guajavae* are slender and sinuous, see Remeikis et al. 2015: figs 1, 7).

#### Description.

**Male** (Figs 21, 30, 31, 34, 35). Forewing length 1.8–2.0 mm; wingspan 4.0–4.5 mm (n = 7). ***Head***: frontal tuft orangish ochre to ochre-brown; collar inconspicuous, comprised of piliform, cream scales; scape yellow cream to pale ochre, with some scattered brown scales; sometimes scape entirely cream, without brown scales, glossy; antenna slightly shorter than length of forewing; flagellum with 27–28 segments, pale grey to dark grey, with little purple iridescence. ***Thorax***, tegula and forewing ochreous cream, sparsely speckled with dark brown scales; forewing with an oblique, postmedian fascia formed by black-brown scales, and with two small, black-brown apical and basal spots (the latter may be absent or inconspicuous in some specimens); fringe cream, fringe line irregular, inconspicuous; on underside, forewing pale grey or cream grey in basal half of wing, pale grey in rest; under fold with a distinct row of special scales, only visible in descaled wings (Figs 34, 35); venation with four distal veins: Rs_3_, Rs_4_, M, and A (Figs 28–31). Hindwing glossy, cream to pale grey; on underside, basal third to half usually cream grey, pale grey in rest, or entire hindwing pale grey; fringe pale grey; venation with two distal veins: Rs and M (Figs 32, 33). ***Legs*** cream to ochre cream; on upper side, foreleg and midleg usually densely covered with dark grey or black-grey scales. Abdomen grey-brown on upper side, cream to pale ochre with some brown scales on underside; anal tufts cream, short, inconspicuous.

**Female** (Figs 22–24, 28, 29, 32, 33). Very similar to male but tends to be slightly darker and larger: forewing length 2.0–2.5 mm; wingspan 4.4–5.4 mm (n = 8). Flagellum with about 25–26 segments. Forewing and hindwing undersides pale grey. Abdominal apex wide, truncated, and without anal tufts (Fig. 24). Otherwise as male.

***Male genitalia*** (Figs 36–57). Capsule much longer (ca 325 μm) than wide (ca 185 μm). Vinculum large; ventral plate of vinculum widely rounded, truncated, without lateral lobes. Tegumen almost truncated or forming an inconspicuous, short, widely bilobed pseuduncus, with many setae on each lobe. Uncus thickened, inverted Y-shaped (Figs 41, 42). Gnathos with short but wide central process and slender lateral arms (Figs 36, 37). Valva (Figs 45, 46) 170–200 μm long, 70–90 μm wide, with long apical process (Fig. 45); transtilla without or with short sublateral processes (Figs 50, 54, 55), and with two large, horn-like processes (Figs 47, 48, 50, 55). Anellus thickened laterally (Figs 44, 49, 51, 55) and ventrally (Figs 55–57), membranous dorsally. Phallus (Figs 38, 40) 70–75 μm long; minimal width 35–50 μm, maximal width at base 70–85 μm, without carina; vesica with an inconspicuous cathrema and plate-like cornutus, and thickened folds which in slides resemble cornuti (Fig. 40).

***Female genitalia*** (Figs 58–64). Total length about 560 μm. Anterior apophyses distally bent inwardly, slightly longer or equal to posterior ones (Figs 58, 64). Vestibulum with a wide, complex sclerite (Fig. 63). Corpus bursae rather small (reduced), without pectinations or signa, oval-shaped (Figs 58, 64). Accessory sac enlarged, equal or longer than corpus bursae; ductus spermathecae wide to slender proximally (see Figs 60, 62), with about three shallow convolutions (Fig. 64) and 2–2.5 large, rounded, plate-like vesicles distally (Figs 59, 61). Abdominal apex wide, truncated.

#### Biology

**Figures 7–12.**
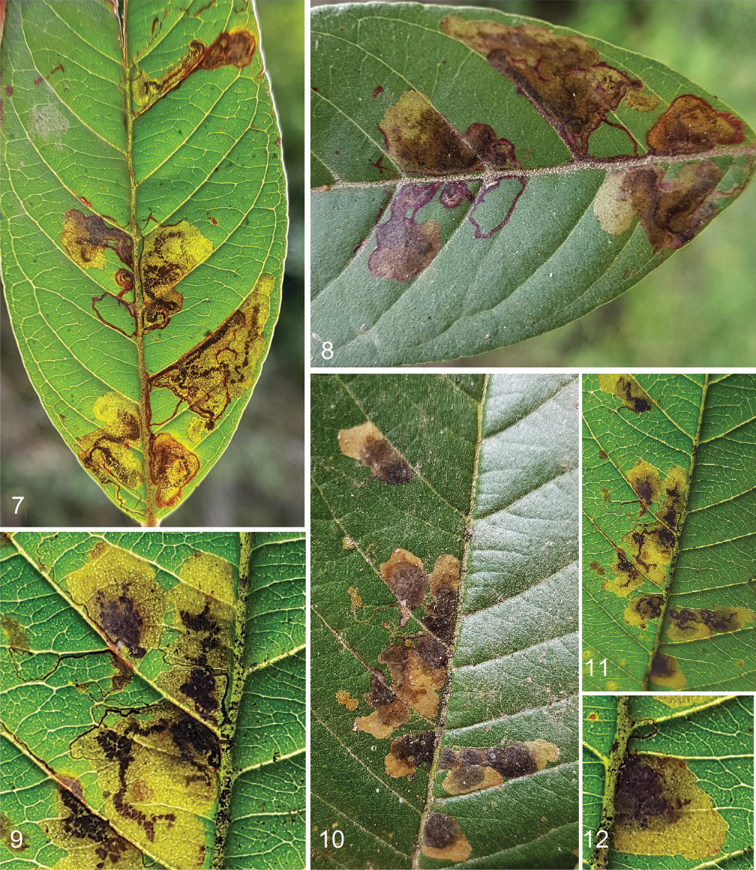
Leaf mines of *Hesperolyra guajavifoliae* Stonis & Vargas sp. nov. on *Psidium guajava* (Myrtaceae), Colombia, Valle del Cauca, Cisneros, 3°46'27"N, 76°44'40"W, 450 m.

**Figures 13–27.**
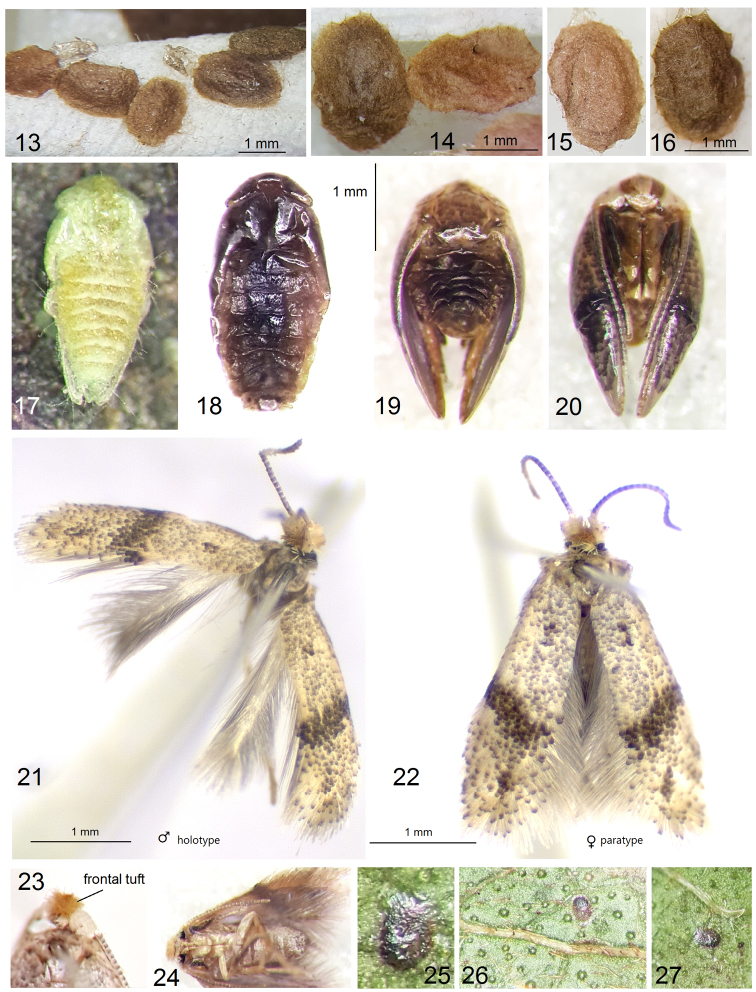
*Hesperolyra guajavifoliae* Stonis & Vargas sp. nov. **13–16** cocoons **17–20** pupae (found dead in cocoons at different stages of development and with various levels of dehydration) **21** male holotype (MPUJ) **22** female paratype **23** frontal tuft, female paratype **24** ventral view, female paratype **25–27** AnEgg on a leaf underside of the host plant *Psidium guajava*.

(Figs 7–20, 25–27). Host plant: *Psidium guajava* (Myrtaceae). Egg (Figs 25, 27) laid singly on underside of leaf; egg case flat, 0.25 mm long (n = 6), shiny, black-grey when filled with frass. Larvae mine leaves in February to early March; based on numerous older, vacant leaf mines, the mining may start as early as late December and be particularly active in January, i.e., during the drier season from late December to February in the exceptionally humid region of western Colombia (see Distribution); voltinism unknown. Larva pale green with a pale brown head and dark green intestine. Leaf mine (Figs 7–12) starts as a slender gallery filled with black frass; later the gallery almost abruptly widens to a blotch with irregularly scattered brown-black or black frass. Pupation occurs outside the leaf mine, possibly in debris or litter, because no cocoons were observed on the host plants. Exit slit on upperside of leaf. Pupation (Figs 17–20) inside cocoon; immature stages will be described elsewhere (Sergio A. Vargas, personal communication). Cocoon (Figs 13–16) 1.9–2.2 mm long, 1.2–1.5 mm wide (n = 9), brown to blackish brown or dark green-brown (slightly paler when vacant and dried), usually with a rather distinct flat rim around the main body (Figs 15, 16). Adults emerged late February to March; moths were not collected at a light trap in localities where the species occurred, therefore, we do not know how readily moths fly to light. Otherwise, biology is unknown.

#### Distribution

(Figs 1–6). So far, this species is known to occur at altitudes from 450 to 850 m on the western slopes of the Andes (Valle del Cauca, western Colombia), bordering with the lowland Choco province. The latter is possibly the most humid area on Earth, where annual rainfall reaches 11,770 mm (Wettest places on Earth 2019) and is equally distributed except for only slight dry season(s) (Figs 2–6).

#### DNA barcode.

We barcoded eight specimens of the type series, but not the holotype; sequences are available in GenBank under voucher/sample IDs MN732873, MN732874, MN732875, MN732876, MN732877, MN732878, MN732879, MN732872.

#### Etymology.

The species name derives from the Latin name of the host plant *guajava*, in combination with the Latin *folium* (a leaf), in reference to the feeding habit of the new species; although the ending -ae here is not correct Latin (van Nieukerken, personal comm.), we preferred to name the species as *guajavifoliae* and not otherwise.

#### Other material examined.

13 ♂, 13 ♀, paratypes: Colombia, Departamento de Valle del Cauca, Municipio de Dagua, Cisneros, 3°46'27"N, 76°44'40"W, 450 m, larva on *Psidium guajava* (Myrtaceae), fieldcard no. SV003, 11 Feb. – 3 Mar. 2019, Jonas R. Stonis and Sergio A. Vargas leg., genitalia slide nos RA1014♂, RA1015♀, RA1016♀, RA1034♀ (MPUJ).

**Figures 28–35.**
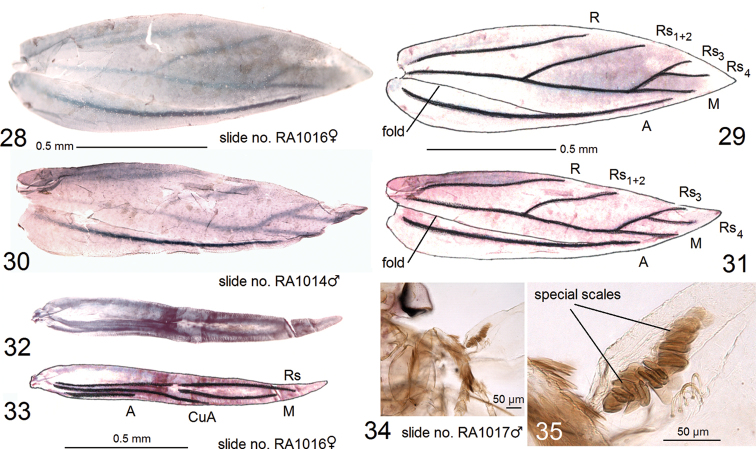
Morphology of *Hesperolyra guajavifoliae* Stonis & Vargas sp. nov. **28** forewing venation, female paratype, slide RA1016 **29** same, enhanced and labelled, with veins reinforced **30** forewing venation, male paratype, slide RA1014 **31** same, enhanced and labelled, with veins reinforced **32** hindwing venation, female paratype, slide RA1016 **33** same, enhanced and labelled, with veins reinforced **34, 35** special scales on descaled male paratype, slide no. RA1017 (MPUJ).

**Figures 36–40.**
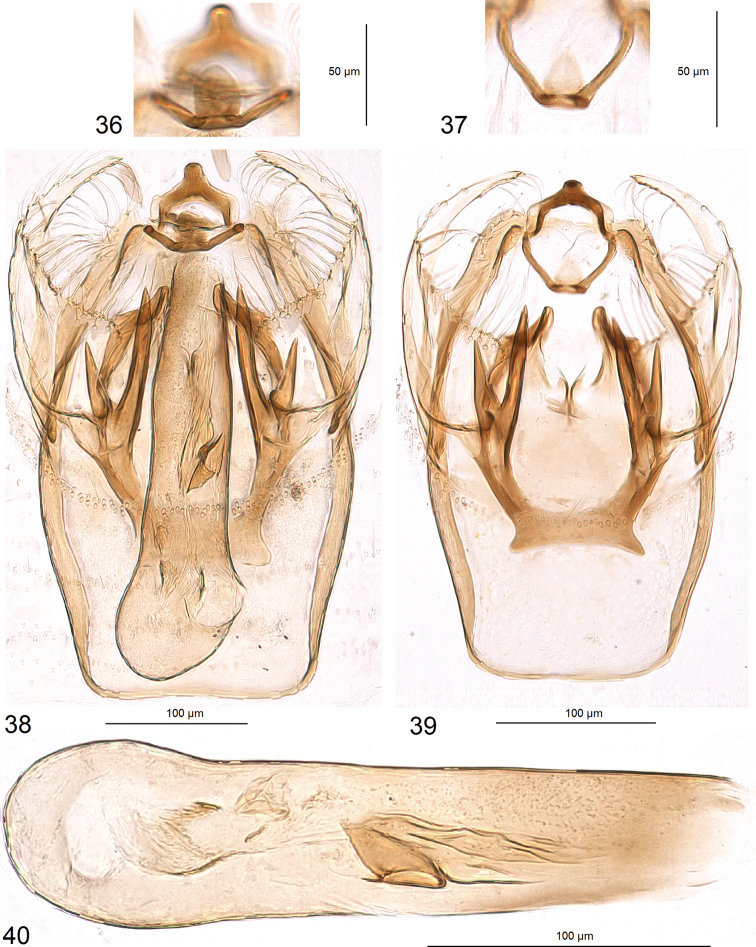
Male genitalia of *Hesperolyra guajavifoliae* Stonis & Vargas sp. nov. **36** gnathos, paratype, genitalia slide no. RA1017 **37** same, holotype, genitalia slide no. RA1033 **38** complete genitalia, paratype, slide no. RA1017 **39** capsule with phallus removed, holotype, genitalia slide no. RA1033 **40** phallus, holotype, genitalia slide no. RA1033 (MPUJ).

**Figures 41–46.**
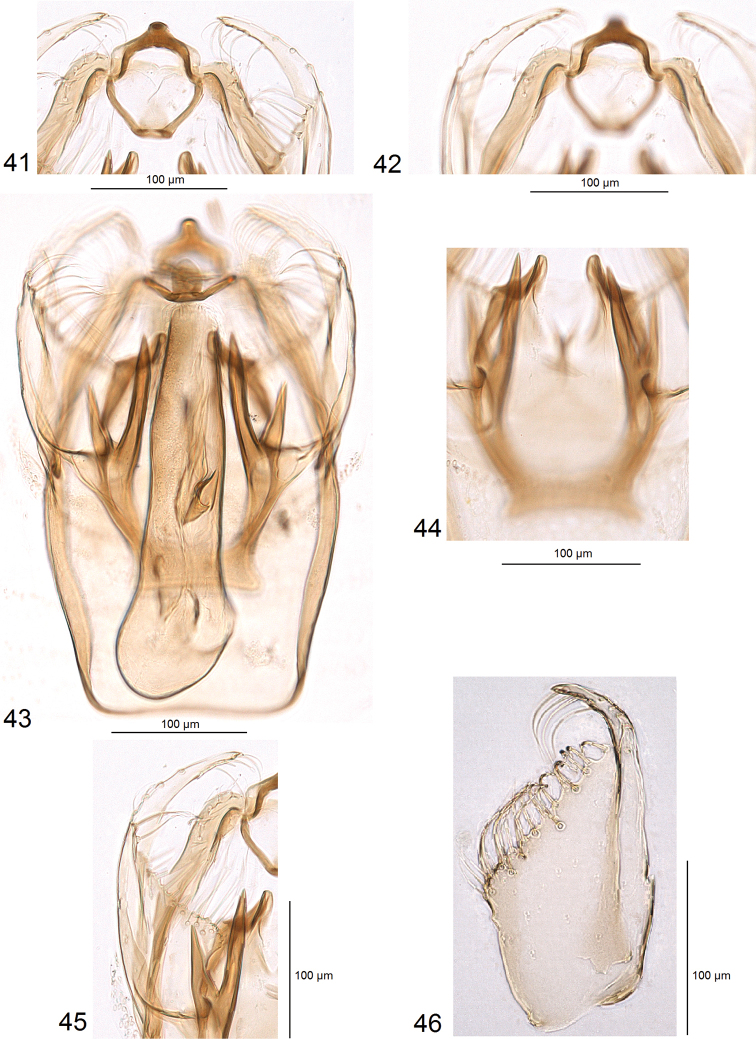
Male genitalia of *Hesperolyra guajavifoliae* Stonis & Vargas sp. nov. **41** tegumen, uncus, and gnathos, holotype, genitalia slide RA1033 **42** same, at different focus **43** complete genitalia, paratype, genitalia slide no. RA1017 **44** anellus and horn-like processes, holotype, genitalia slide RA1033 **45** valva, holotype, genitalia slide no. RA1033 **46** same, paratype, genitalia slide no. RA1018 (MPUJ).

**Figures 47–57.**
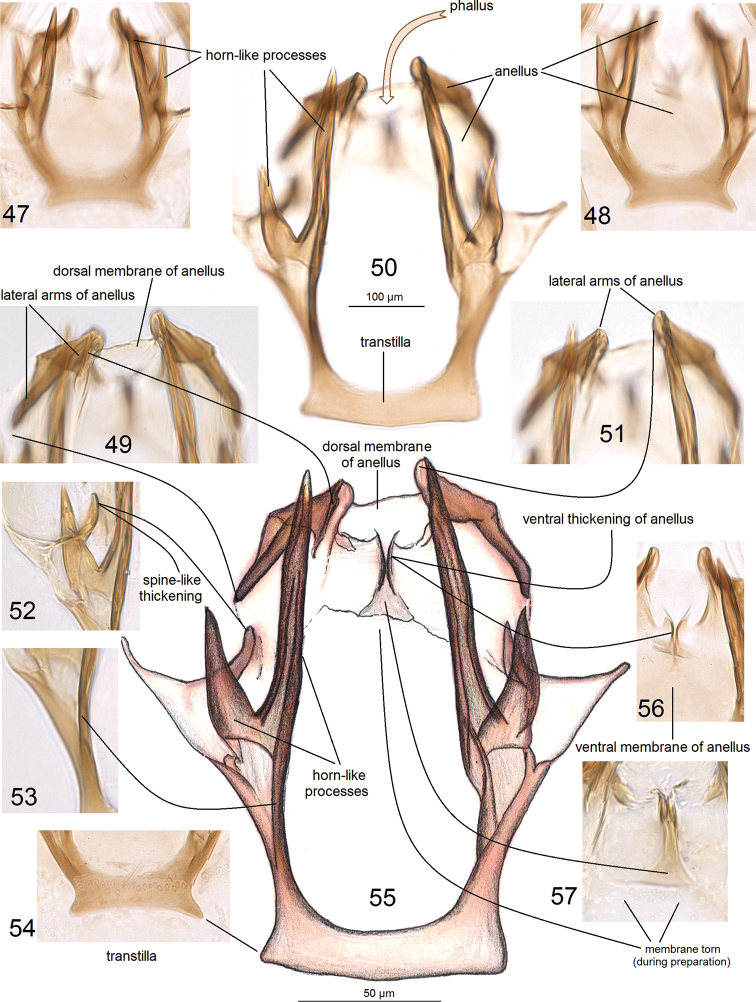
Male genitalia of *Hesperolyra guajavifoliae* Stonis & Vargas sp. nov. Details of morphology **47, 48, 54, 56** holotype, genitalia slide no. RA1033 **49–53, 55, 57** paratype, genitalia slide no. RA1018 (MPUJ).

**Figures 58–64.**
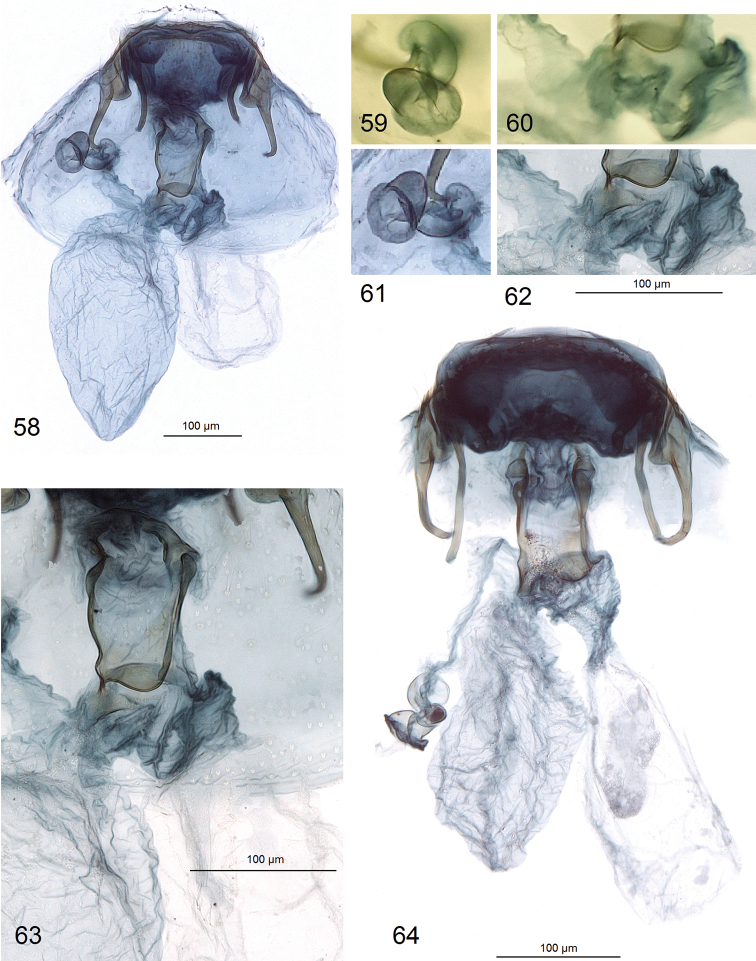
Female genitalia of *Hesperolyra guajavifoliae* Stonis & Vargas sp. nov. **58–63** paratype, genitalia slide no. RA1015 **64** same, genitalia slide no. RA1034 (MPUJ).

**Figures 65–76.**
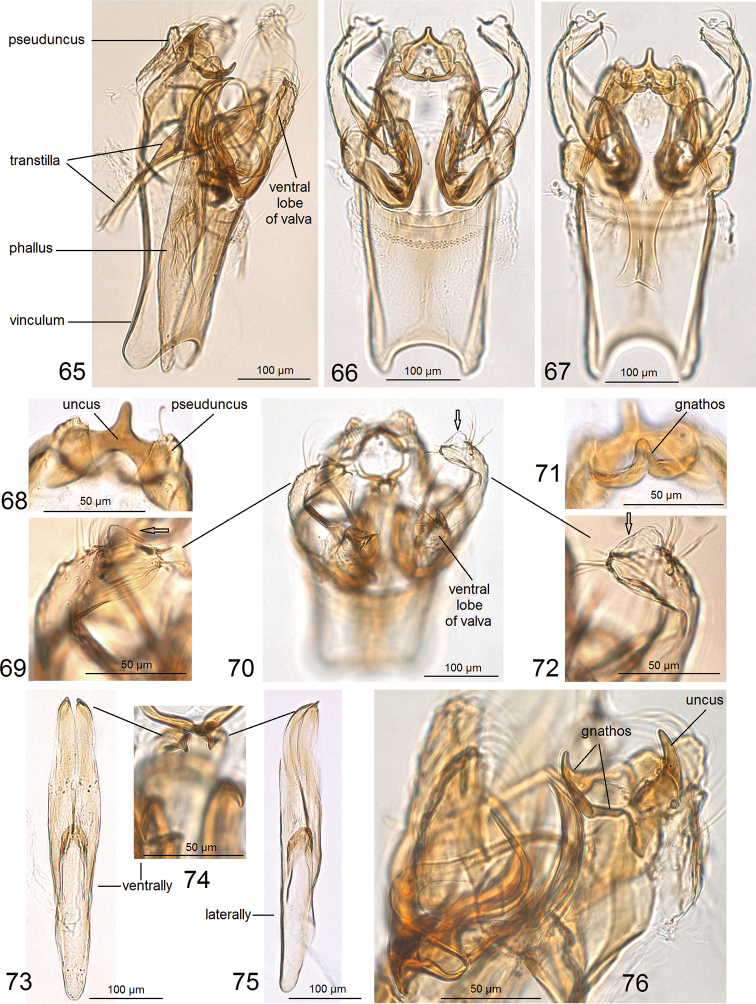
Details of male genitalia of *Hesperolyra diskusi* Puplesis & Robinson, paratype, genitalia slide no. AD989 (ZMUC).

**Figures 77–86.**
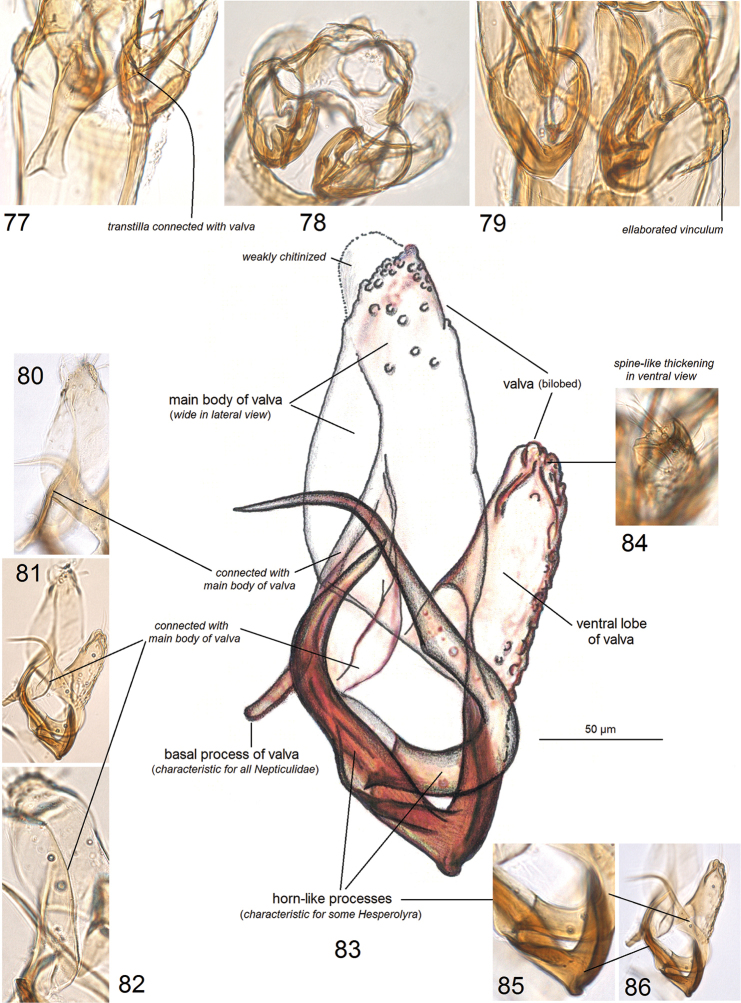
Details of male genitalia of *Hesperolyra diskusi* Puplesis & Robinson **77–79, 84** paratype, genitalia genitalia slide no. AD989 (NHMUK) **80–83, 85, 86** paratype, genitalia slide no. AD962 (ZMUC).

## Discussion

In the first review of the Neotropical Nepticulidae (Puplesis and Robinson 2000) we noticed that some nepticulid species from Belize and Colombia looked different, mostly because of the long transtilla and horn-like processes in the male genitalia. We hypothesized that these species could belong to *Fomoria* Beirne, but the observed difference in the forewing venation in comparison to *Fomoria* (Puplesis and Robinson 2000: fig. 64) was incorrectly hypothesized to be a case of partial venational reduction. Later, a formal species group within *Fomoria* was erected for species with these male genitalic and forewing venational characters (Puplesis et al. 2002b). In the most recent review and global catalogue (van Nieukerken et al. 2016a, 2016b), the *molybditis* group was excluded from *Fomoria* and erected as a separate genus.

For this study, we re-examined the type species of *Hesperolyra*, *H. diskusi* (Puplesis & Robinson, 2000), that is characterized by a complex morphology of the male genitalia (Figs 65–86). The horn-like processes are the most remarkable genitalic features of this species. Characterization of *H. diskusi* was provided in the original description (Puplesis and Robinson 2000) and later in the redescription by van Nieukerken et al. (2016b). In the current study, we found two, not three, large processes (Figs 83–86), and observed that they are attached to the valva (Figs 78–83), not the anellus as was supposed earlier (van Nieukerken et al. 2016b); we did not observe the presence of an anellus. We also found that the transtilla in *H. diskusi* is more flexible and movable in comparison to most Nepticulidae that possess a transtilla; it can be lifted slightly dorsally at an angle even if the valvae are fixed (Figs 65, 77), and the connections of the transtilla to the valva are unusually weak, easy to break since they are supported by slender, little chitinized arms (Fig. 67).

We provide photographic documentation of the genital structures at different angles (Figs 65–86); they, including the spine-like thickening on the ventral lobe of the valva (Fig. 84) or the ventrally-bent spines of the phallus (Fig. 74), are usually not available for observation or appear different in permanent mounts. After our examination, we became convinced that none of the large, horn-like processes are carinal processes of the phallus.

Upon comparison of the male genitalia, we found that *Hesperolyra guajavifoliae* sp. nov. fundamentally differs in morphology from *H. diskusi*. We discovered that in *H. guajavifoliae* the horn-like processes are connected not with the valva itself, but are fused with the transtilla (Figs 50, 55), and, in contrast to *H. diskusi*, the anellus is present in the male genitalia. We found that the anellus is comprised of a weakly chitinized dorsal membrane (Figs 49, 55), strongly thickened lateral arms (Figs 49, 51, 55), and a ventral membrane with an elaborate thickening (Figs 55–57) surrounding the phallus from all sides (Fig. 50, best seen in Figs 38, 43).

**Figure 87.**
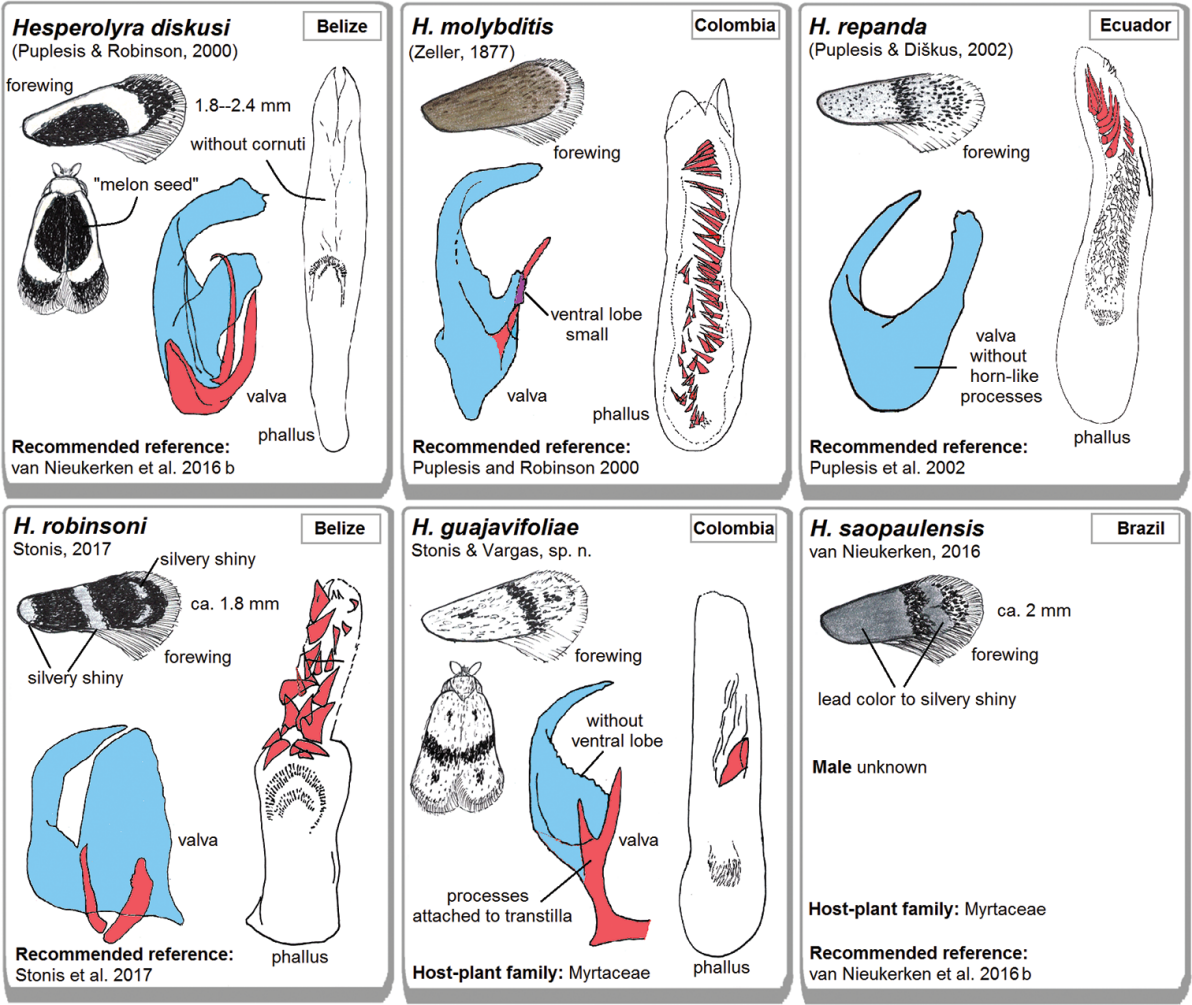
A pictorial tool for quick diagnostics of the currently known *Hesperolyra* species. Note: the morphological structures are drawn at different scales.

Currently, there are six species of *Hesperolyra* distributed from Central America (Belize) to the Atlantic coast of Brazil (Fig. 1); the species from Brazil was described from a female only (van Nieukerken et al. 2016b). All species are distinctive; therefore, diagnostics of *Hesperolyra* species, including the Brazilian *H. saopaulensis*, is clear (see Fig. 87). However, it also raises some questions: do all the species really belong to the same genus, and what is their relationship to other genera?

The wing venation of *Hesperolyra guajavifoliae* sp. nov. (Figs 28–33) is almost identical to *H. saopaulensis* (see van Nieukerken et al. 2016b: fig. 116) and similar to *H. diskusi* (see Puplesis and Robinson 2000: fig. 64); the venation of remaining species is unstudied. It is important to note that unique, special scales hidden under the forewing fold are characteristic of both *H. diskusi* and *H. guajavifoliae* sp. nov. We have concluded that *Hesperolyra* most likely represents a separate, monophyletic taxon, characterized by a wide forewing, with more or less uniform, but unique, simplified venation, extended, lyre-shaped transtilla, elaborate valva, and the presence of horn-like processes in the male genitalia, and possibly the feeding on Myrtaceae (host plant known for only two species). *Hesperolyra* was also supported by a multi-gene molecular analysis by Doorenweerd et al. (2016), that grouped it with *Neotrifurcula* van Nieukerken and *Bohemannia* Stainton. *Neotrifurcula* was subsequently synonymized with *Glaucolepis* Braun (Stonis et al. 2017).

During our study, ten sequences of 657 bp and 1 sequence of 609 bp of the mtDNA COI gene belonging to three Nepticulidae and one Opostegidae species were successfully obtained (Table 1). These data were supplemented by the sequences of other species downloaded from the BOLD website (Table 2). In these sequences, 184 parsimony-informative sites were detected. The overall mean distance between analysed species estimated using the same mtDNA sequence was 14.8 ± 1.0. The interspecific pairwise distances between the pairs of the studied species varied from 5.6 ± 1.0% (between *Etainia albibimaculella* (Larsen) and *E. capesella* (Puplesis)), and 22.1 ± 2.5% (between *H. guajavifoliae* sp. nov. and *Pseudopostega* sp.). The smallest interspecific distance from *H. guajavifoliae* sp. nov. to any other species was 15.3 ± 1.9% (i.e., between *H. guajavifoliae* sp. nov. and *Fomoria eriki*) (Table 2). This indicates that COI can be used as a useful diagnostic tool for the identification of this new species. On the other hand, intraspecific divergence in *H. guajavifoliae* sp. nov. has not been observed yet; however, all studied specimens were from the same locality, and additional specimens from different localities would certainly enrich our knowledge about divergence within the species.

**Figures 88–90.**
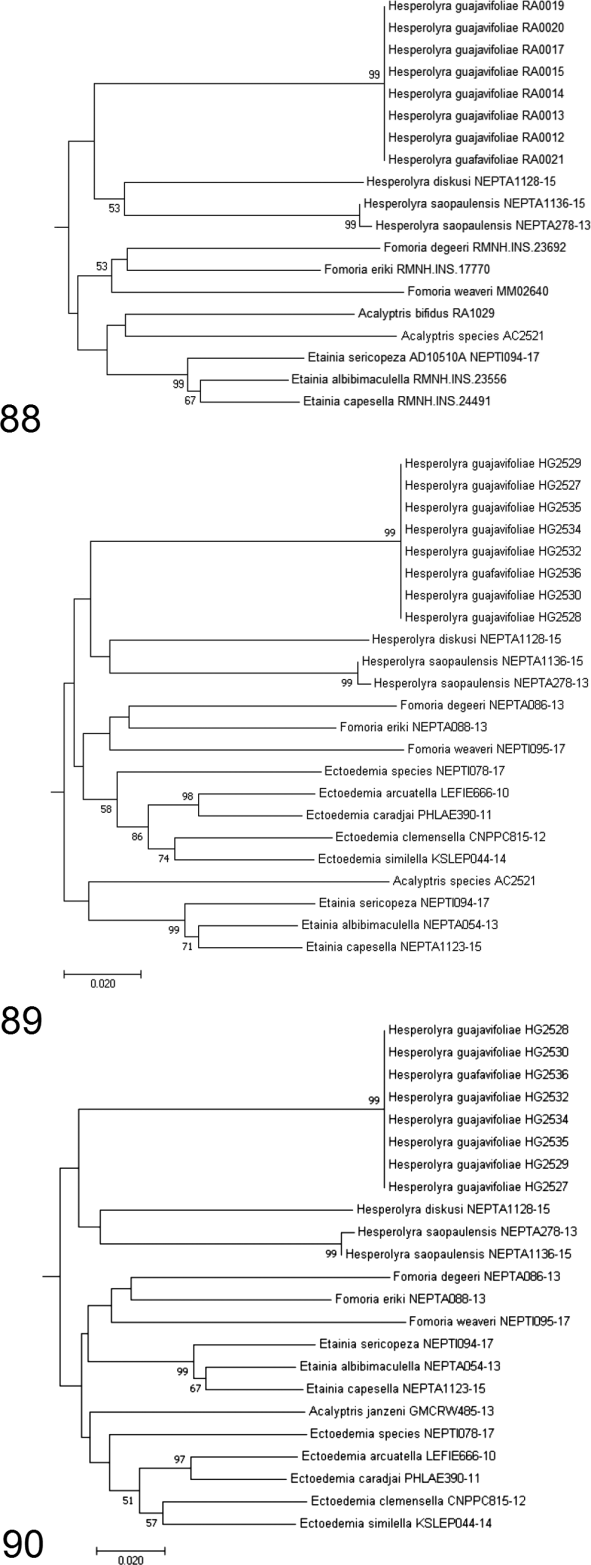
Fragments of different versions of Neighbour-Joining tree of *Hesperolyra* and other Nepticulidae genera (the full molecular phylogeny will be published elsewhere; a general phylogeny of Nepticulidae is not presented or discussed here). The divergence was calculated using the Kimura 2-parameter model based on 657 bp mtDNA COI sequences. Bootstrap values below 50 are not shown. *Pseudopostega* sp. (Opostegidae) was used as outgroup.

Depending on the combination of species set, several versions of the Neighbour-Joining tree with different topology were obtained; some of them are presented in Figs 88–90. In our preliminary analysis using only the COI barcode fragment, *Hesperolyra* always appeared as a separate clade. *Hesperolyra guajavifoliae* sp. nov. always clustered at a distance from *H. diskusi* + *H. saopaulensis* (Fig. 88). In most of our numerous, different attempts, the *Hesperolyra* clade consistently grouped either with *Fomoria* + *Ectoedemia* (Figs 89), or *Fomoria* + [*Acalyptris* + *Etainia*] (Fig. 88), or [*Fomoria* + *Etainia*] + [*Acalyptris* + *Ectoedemia*] (Fig. 90), or even only with *Fomoria*, but never with *Glaucolepis* or *Bohemannia*, or other genera, as presented in Doorenweerd et al. (2016). We found that the relationships between approximately half of the clades remain unsupported according to bootstrapping results; however, according to Nieukerken et al. (2012), bootstrap support values for the Neighbour-Joining similarity tree are not necessary. NJ trees are never robust due to the nature of the method; therefore, adding bootstrap supports to indicate the robustness does not add much. NJ trees are useful for indicating pairwise differences between clusters (of species) and estimating whether COI can be used as a diagnostic marker, what has been shown in the case of *H. guajavifoliae* sp. nov. Although our data are far from complete, the tendency of *Hesperolyra* to group with *Fomoria* or other genera causes us to re-evaluate their relationships; i.e., it may be possible that *Hesperolyra* is related to these taxa. It is interesting to note that, unexpectedly, *Acalyptris* most often clustered with *Etainia* (e.g., Figs 88, 90); this was also found by other workers (Doorenweerd et al. 2016).

Our molecular analysis did not show a close relationship between the guava-feeding *Hesperolyra guajavifoliae* sp. nov. and other Myrtaceae-feeding Nepticulidae, including the South American guava-feeding nepticulid species, *Ozadelpha guajavae*, which was recently barcoded by us; the sequence is available in the BOLD database: ADH4024.

So far, *Hesperolyra guajavifoliae* sp. nov. is the only Nepticulidae pest discovered in western Colombia. However, during our fieldwork we observed a couple of old leaf mines on guava with a wider gallery that did not extend into an obvious blotch at the final stage of development. Although these differently looking leaf mines may belong to *Ozadelpha guajavae*, there is no confirmed evidence that both species *H. guajavifoliae* sp. nov. and *O. guajavae* occur together in western Colombia.

**Table 2.** Pairwise distances between sequences. The number of base substitutions per site between sequences (%) are shown; standard error estimates (%) are shown above the diagonal and were obtained by a bootstrap procedure (10,000 replicates). Analyses were conducted using the Kimura 2-parameter model.

		1	2	3	4	5	6	7	8	9	10	11	12	13	14	15	16	17	18	19	20	21	22	23	24	25	26
**1**	*Hesperolyra guafavifoliae* HG2536		0.000	0.000	0.000	0.000	0.000	0.000	0.000	0.021	0.020	0.020	0.023	0.019	0.023	0.022	0.021	0.023	0.019	0.020	0.019	0.020	0.021	0.019	0.019	0.020	0.025
**2**	*H. guajavifoliae* HG2527	0.000		0.000	0.000	0.000	0.000	0.000	0.000	0.021	0.020	0.020	0.023	0.019	0.023	0.022	0.021	0.023	0.019	0.020	0.019	0.020	0.021	0.019	0.019	0.020	0.025
**3**	*H. guajavifoliae* HG2528	0.000	0.000		0.000	0.000	0.000	0.000	0.000	0.021	0.020	0.020	0.023	0.019	0.023	0.022	0.021	0.023	0.019	0.020	0.019	0.020	0.021	0.019	0.019	0.020	0.025
**4**	*H. guajavifoliae* HG2529	0.000	0.000	0.000		0.000	0.000	0.000	0.000	0.021	0.020	0.020	0.023	0.019	0.023	0.022	0.021	0.023	0.019	0.020	0.019	0.020	0.021	0.019	0.019	0.020	0.025
**5**	*H. guajavifoliae* HG2530	0.000	0.000	0.000	0.000		0.000	0.000	0.000	0.021	0.020	0.020	0.023	0.019	0.023	0.022	0.021	0.023	0.019	0.020	0.019	0.020	0.021	0.019	0.019	0.020	0.025
**6**	*H. guajavifoliae* HG2532	0.000	0.000	0.000	0.000	0.000		0.000	0.000	0.021	0.020	0.020	0.023	0.019	0.023	0.022	0.021	0.023	0.019	0.020	0.019	0.020	0.021	0.019	0.019	0.020	0.025
**7**	*H. guajavifoliae* HG2534	0.000	0.000	0.000	0.000	0.000	0.000		0.000	0.021	0.020	0.020	0.023	0.019	0.023	0.022	0.021	0.023	0.019	0.020	0.019	0.020	0.021	0.019	0.019	0.020	0.025
**8**	*H. guajavifoliae* HG2535	0.000	0.000	0.000	0.000	0.000	0.000	0.000		0.021	0.020	0.020	0.023	0.019	0.023	0.022	0.021	0.023	0.019	0.020	0.019	0.020	0.021	0.019	0.019	0.020	0.025
**9**	*H. diskusi* NEPTA1128-15	0.174	0.174	0.174	0.174	0.174	0.174	0.174	0.174		0.018	0.018	0.022	0.019	0.023	0.022	0.019	0.023	0.019	0.020	0.021	0.018	0.018	0.019	0.018	0.019	0.023
**10**	*H. saopaulensis* NEPTA1136-15	0.159	0.159	0.159	0.159	0.159	0.159	0.159	0.159	0.142		0.002	0.021	0.020	0.021	0.022	0.019	0.022	0.020	0.021	0.021	0.020	0.019	0.017	0.019	0.018	0.022
**11**	*H. saopaulensis* NEPTA278-13	0.164	0.164	0.164	0.164	0.164	0.164	0.164	0.164	0.146	0.003		0.021	0.020	0.021	0.022	0.019	0.022	0.020	0.021	0.021	0.020	0.019	0.017	0.019	0.018	0.022
**12**	*Fomoria degeeri* NEPTA086-13	0.203	0.203	0.203	0.203	0.203	0.203	0.203	0.203	0.190	0.172	0.172		0.017	0.022	0,020	0.019	0.024	0.018	0.018	0.019	0.018	0.017	0.019	0.018	0.018	0.023
**13**	*F. eriki* NEPTA088-13	0.153	0.153	0.153	0.153	0.153	0.153	0.153	0.153	0.155	0.165	0.169	0.134		0.019	0.021	0.019	0.021	0.017	0.018	0.017	0.017	0.017	0.019	0.018	0.017	0.024
**14**	*F. weaveri* NEPTI095-17	0.194	0.194	0.194	0.194	0.194	0.194	0.194	0.194	0.192	0.172	0.172	0.175	0.143		0.023	0.021	0.024	0.020	0.021	0.020	0.021	0.022	0.021	0.023	0.020	0.024
**15**	*Acalyptris bifidus* AB2517	0.184	0.184	0.184	0.184	0.184	0.184	0.184	0.184	0.177	0.187	0.192	0.164	0.171	0.188		0.019	0.020	0.016	0.016	0.018	0.020	0.019	0.019	0.019	0.020	0.021
**16**	*A. janzeni* GMCRW485-13	0.173	0.173	0.173	0.173	0.173	0.173	0.173	0.173	0.154	0.157	0.162	0.155	0.151	0.167	0.141		0.020	0.016	0.017	0.017	0.016	0.016	0.014	0.015	0.018	0.022
**17**	*Acalyptris* sp. AC2521	0.192	0.192	0.192	0.192	0.192	0.192	0.192	0.192	0.187	0.179	0.185	0.199	0.168	0.194	0.150	0.164		0.019	0.019	0.019	0.020	0.020	0.021	0.019	0.023	0.025
**18**	*Etainia albibimaculella* NEPTA054-13	0.157	0.157	0.157	0.157	0.157	0.157	0.157	0.157	0.157	0.173	0.178	0.148	0.127	0.165	0.121	0.121	0.148		0.010	0.011	0.015	0.014	0.016	0.014	0.018	0.023
**19**	*E. capesella* NEPTA1123-15	0.165	0.165	0.165	0.165	0.165	0.165	0.165	0.165	0.163	0.175	0.180	0.144	0.142	0.168	0.121	0.128	0.147	0.056		0.011	0.014	0.014	0.016	0.015	0.018	0.023
**20**	*E. sericopeza* NEPTI094-17	0.156	0.156	0.156	0.156	0.156	0.156	0.156	0.156	0.172	0.178	0.183	0.155	0.135	0.154	0.135	0.130	0.148	0.067	0.066		0.015	0.016	0.017	0.016	0.018	0.022
**21**	*Ectoedemia arcuatella* LEFIE666-10	0.172	0.172	0.172	0.172	0.172	0.172	0.172	0.172	0.150	0.168	0.173	0.148	0.127	0.169	0.154	0.120	0.166	0.111	0.112	0.123		0.010	0.015	0.013	0.015	0.022
**22**	*E. caradjai* PHLAE390-11	0.179	0.179	0.179	0.179	0.179	0.179	0.179	0.179	0.150	0.159	0.163	0.140	0.125	0.177	0.148	0.126	0.162	0.107	0.112	0.128	0.059		0.014	0.012	0.014	0.021
**23**	*E. clemensella* CNPPC815-12	0.161	0.161	0.161	0.161	0.161	0.161	0.161	0.161	0.157	0.143	0.143	0.158	0.148	0.173	0,150	0.100	0.174	0.125	0.126	0.141	0.106	0.096		0.012	0.016	0.022
**24**	*E. similella* KSLEP044-14	0.163	0.163	0.163	0.163	0.163	0.163	0.163	0.163	0.143	0.162	0.166	0.153	0.138	0.181	0.150	0.112	0.159	0.113	0.112	0.123	0.085	0.081	0.081		0.017	0.023
**25**	*Ectoedemia* sp. NEPTI078-17	0.165	0.165	0.165	0.165	0.165	0.165	0.165	0.165	0.154	0.139	0.144	0.149	0.131	0.159	0.159	0.135	0.189	0.138	0.137	0.143	0.112	0.100	0.116	0.120		0.022
**26**	*Pseudopostega* sp. PC2516	0.221	0.221	0.221	0.221	0.221	0.221	0.221	0.221	0.204	0.187	0.190	0.215	0.220	0.215	0.173	0.185	0.204	0.197	0.197	0.190	0.189	0.175	0.194	0.199	0.182	

## Contributions the research

Contributions to this research are as follows: JRS: concept and design of the research and fieldwork, discovery and rearing of the adults from the mining larvae, photographic documentation of leaf mines and habitats; writing the manuscript and technical preparation of all plates of illustrations, and discussion on results of the molecular research and diagnostics of *Hesperolyra*; AR: preparation of the material collected in Colombia in 2019, dissection and photographic documentation of *H. guajavifoliae* sp. nov., molecular research, discussion on diagnostics of *Hesperolyra* and general Nepticulidae phylogeny, and writing of comments on the results of molecular research; AD: dissection and photographic documentation of type species (*H. diskusi*), discussion on morphology and diagnostics of *Hesperolyra*, compiling of list of cited references; SO: molecular research, discussion on molecular Nepticulidae phylogeny, writing of comments on the results of DNA research, and deposition of sequences in GenBank; SA: fieldwork in Colombia, assistance in rearing some *H. guajavifoliae* sp. nov., obtaining of research permits (together with Igor Dimitri Forero Fuentes, see Acknowledgements), various contributions to the new species description, specimen deposition at MPUJ; MAS: manuscript writing, scientific expertise of the data, elaboration of the concept, and discussion on the results.

## Supplementary Material

XML Treatment for Hesperolyra guajavifoliae
